# Evaluation of a solid matrix for collection and ambient storage of RNA from whole blood

**DOI:** 10.1186/1472-6890-14-22

**Published:** 2014-05-13

**Authors:** Heng Tao, Philip Beineke, Bing Li, William Alberts, Steven Rosenberg, Erik Kvam, James A Wingrove

**Affiliations:** 1 CardioDx, Inc., 2500 Faber Place, Palo Alto, CA 94303, USA; 2 GE Global Research, One Research Circle, K1 5D29 Niskayuna NY 12309, USA

## Abstract

**Background:**

Whole blood gene expression-based molecular diagnostic tests are becoming increasingly available. Conventional tube-based methods for obtaining RNA from whole blood can be limited by phlebotomy, volume requirements, and RNA stability during transport and storage. A dried blood spot matrix for collecting high-quality RNA, called RNA Stabilizing Matrix (RSM), was evaluated against PAXgene® blood collection tubes.

**Methods:**

Whole blood was collected from 25 individuals and subjected to 3 sample storage conditions: 18 hours at either room temperature (baseline arm) or 37°C, and 6 days at room temperature. RNA was extracted and assessed for integrity by Agilent Bioanalyzer, and gene expression was compared by RT-qPCR across 23 mRNAs comprising a clinical test for obstructive coronary artery disease.

**Results:**

RSM produced RNA of relatively high integrity across the various tested conditions (mean RIN ± 95% CI: baseline arm, 6.92 ± 0.24; 37°C arm, 5.98 ± 0.48; 6-day arm, 6.72 ± 0.23). PAXgene samples showed comparable RNA integrity in both baseline and 37°C arms (8.42 ± 0.17; 7.92 ± 0.1 respectively) however significant degradation was observed in the 6-day arm (3.19 ± 1.32). Gene expression scores on RSM were highly correlated between the baseline and 37°C and 6-day study arms (median r = 0.96, 0.95 respectively), as was the correlation to PAXgene tubes (median r = 0.95, p < 0.001).

**Conclusion:**

RNA obtained from RSM shows little degradation and comparable RT-qPCR performance to PAXgene RNA for the 23 genes analyzed. Further development of this technology may provide a convenient method for collecting, shipping, and storing RNA for gene expression assays.

## Background

A variety of physiological changes and disease states can alter gene expression in peripheral blood cells. Measuring the level of specific RNA species in the bloodstream has proven to be a powerful tool for monitoring disease occurrence and stratifying patient risk for a variety of oncological, neurological, immunological, and cardiovascular disorders [1-5].

Blood is a plentiful and highly accessible fluid that can be sampled through minimally invasive techniques. However, a number of physiological and environmental factors can reduce the quality of blood samples and blood RNA when maintained *ex vivo*. First, gene expression artifacts are known to occur rapidly after phlebotomy collection unless blood cell metabolism is immediately arrested in some manner such as freezing or chemical lysis [6]. Second, RNA species are highly sensitive to enzymatic degradation from ubiquitous ribonucleases (RNases) that are endemic to biological samples and environmental surfaces. Lastly, RNA is chemically unstable and prone to self-hydrolysis through trans-esterification reactions catalyzed by temperature, alkaline pH, and water [7]. These preanalytical issues have traditionally demanded that RNA be isolated and maintained at low temperatures immediately after blood collection.

For many research and diagnostic applications, immediate RNA purification is inconvenient or impossible, and maintaining RNA samples at low temperatures adds significant costs. Several liquid preservatives have been developed to stabilize RNA immediately upon blood collection and reduce dependencies on refrigeration, including PAXgene® and TEMPUS® blood collection tubes [8,9]. However, these tube-based methods afford a limited window for RNA stabilization at ambient temperature (3 days or 5 days at 18-25°C, respectively), require a trained phlebotomist to implement, and involve careful mixing of blood in an excess volume of liquid preservative. As a consequence, tube-based collection formats are prone to poor RNA quality if filled imperfectly or incorrectly, and are not recommended for use in uncontrolled environments such as field collection or standard shipping [10]. The volumetric requirements of PAXgene and TEMPUS (2.5 mL or 3 mL whole blood, respectively) are also not suitable for applications involving infants and small animals [11,12]. These factors led us to test the feasibility of developing a solid matrix for simultaneous collection and preservation of high-quality RNA in whole blood samples, similar to FTA® technology. FTA is a filter paper-based product line that streamlines sample handling and nucleic acid preservation by permitting sample collection and stabilization steps at the point-of-collection in a substantially dry state [13].

In this study, we evaluate a dried blood spot matrix optimized for collecting and preserving high-quality RNA, termed RNA Stabilizing Matrix (RSM), and compare RNA integrity and gene expression levels relative to PAXgene blood collection tubes drawn in parallel. Gene expression was compared across 23 target messenger RNAs comprising the Corus® CAD test, a clinically-validated test that identifies the likelihood of obstructive coronary disease [4,14,15]. We demonstrate that comparable results are obtained between RSM filter paper and PAXgene blood collection tubes under overnight sample storage conditions, and RSM performance exceeds PAXgene results with longer-term room temperature storage. The features offered by this improved dried blood spot technology may increase patient access and enable cost-effective sample transport in uncontrolled environments while maintaining high sample integrity.

## Methods

### RSM preparation

RSM is a filter paper-based matrix containing a proprietary reagent formulation for lysing blood cells and stabilizing RNA. Test samples were prepared for this study using a GE Modular Membrane Modification (GEM3) pilot manufacturing line at GE Global Research.

### Rat blood collection and dried blood spot extraction

Male Sprague–Dawley rats (180-250 g) were purchased from Charles River Laboratories. The experimental protocol of this study was approved by the IACUC at General Electric Global Research (Niskayuna, NY), which is an AAALAC, USDA, and OLAW accredited facility. Rats were housed in standard cages (Alternative Design Manufacturing & Supply Inc.), and throughout the study were provided with *ad libitum* access to standard commercial feed (Lab Diet; Purina Mills) and water. All animals were maintained on a 12-hr:12-hr light: dark cycle in rooms with controlled temperature (approximately 24°C) and humidity (approximately 40%). Whole blood was collected via pipette directly from a 26 gauge catheter placed in the tail vein of anaesthetized rats. Fifty microliter aliquots were transferred without anticoagulant and spotted onto chemically-treated and untreated filter papers. Blood spots were dried and maintained at ambient lab temperature for 11 days in a desiccator cabinet (~20% relative humidity). Two sample discs were punched from each dried blood spot using a 7 mm Harris Uni-Core punch (Fisher Scientific) treated with 15 μl PK solution (4 mg/ml proteinase K +0.5%SDS) per punch. Sample discs were transferred into a 1.5 ml microcentrifuge tube containing 350 μl of extraction solution (RLT buffer with 1% β-mercaptoethanol, Qiagen) and incubated for 20 min at 40°C on a Thermomixer (Eppendorf) at 700 rpm. After incubation, RNA was purified from 350 μl of eluate using QIAamp RNA Blood Mini kit (Qiagen) and eluted in 40 μl of nuclease-free water.

### Human blood collection and application onto RSM paper

The whole blood used in the study was collected from donors who had granted written consent. Ethics approval for this study was obtained from the Western Institutional Review Board (Protocol #20090362). Venous blood samples were collected via phlebotomy into EDTA or heparin coated blood tubes (Fisher Scientific). Immediately after blood drawing and mixing, 50 μl of blood was pipetted from the blood tubes and applied onto one piece of RSM paper by dispensing in a circular motion within the spotted circle. The samples were allowed to dry at room temperature for ~2 hours and then placed back into the original Mylar pouch with fresh desiccant and stored at three different conditions: overnight (18 hours) at room temperature (~25°C), 6 days at room temperature or overnight (18 hours) at 37°C .

### Human RNA extraction from RSM paper

Four to five 6 mm sample discs were prepared from each dried blood spot by using a 6 mm Harris Uni-Core punch (Fisher Scientific). The sample discs were placed on parafilm and 15 μl PK solutions (4 mg/ml proteinase K +0.5%SDS) was added onto each disc and incubated at room temperature for 15 minutes. Discs from 2 blood spots were placed into 1.5 ml microcentrifuge tubes containing 800 μl of extraction solution (RLT buffer with 1% β-mercaptoethanol, Qiagen) and incubated for 30 min at 37°C on a Thermomixer (Eppendorf) at 600 rpm. After incubation, 700 μl of eluate was transferred to a new microcentrifuge tube and mixed with 400 μl of isopropanol and 10 μl SPRI beads from the Agencourt RNAdvance Blood kit (Beckman Coulter Genomics). RNA was then extracted following the manufacturer’s instruction; genomic DNA removal was performed using the Ambion DNase I kit (Ambion) at 37°C for 10 min.

### PAXgene RNA blood collection and RNA extraction

Human whole blood samples were collected in PAXgene® RNA Blood Tubes according to the manufacturer’s instructions (PreAnalytix). RNA was isolated by means of a magnetic bead based approach using a modified version of the Agencourt RNAdvance Blood kit (Beckman Coulter Genomics) and the Hamilton STAR automated liquid handler (Hamilton). Extraction was performed in 96-well plates containing 400 μl of whole blood per well; genomic DNA removal was performed using the Ambion DNase I kit (Ambion) at 37°C for 10 min.

### Internal PAX pool blood control

Approximately 2.5 mL of blood was collected in PAXgene® RNA Blood Vacutainer Tubes from consented donors (Western IRB Protocol #20090362). The blood/PAXgene reagent mixture from donor tubes were pooled together. After pooling and mixing, each pool was distributed into 1.5 ml aliquots and stored at -20°C.

### RNA analysis

Purified RNA was quantified by absorbance at 260 nm using the NanoDrop 8000 (Thermo Scientific). RNA integrity was assessed by Agilent 2100 Bioanalyzer using the Eukaryote total RNA 6000 Pico Assay kit according to the manufacturer’s instructions (Agilent Technologies, Palo Alto, CA). The RNA integrity numbers (RIN) were calculated using the Agilent 2100 Expert Software (RIN = 1; lowest RNA quality to RIN = 10; highest RNA quality).

### Reverse transcription

RNA was reverse-transcribed to cDNA using the High Capacity Reverse Transcription Kit (Life Technologies). The RNA extracted from PAXgene tubes was adjusted to 6 ng/μl and cDNA samples diluted to a RNA equivalent of 1 ng/μl for downstream processing. All available RNA extracted from RSM paper (estimated ~200 ng) was used in cDNA reaction without mass normalization.

### RT-qPCR gene expression

The manufacture of commercial GES RT-qPCR assay plates is described elsewhere [16]. RT-qPCR reactions were performed by addition of 2 μL of cDNA sample onto the GES plates. All RT-qPCR reactions were run using the Light Cycler 480 II (Roche) using the following cycling condition: 50°C 2 minutes, 95°C 10 minutes and 45 cycles of 95°C 15 seconds and 60°C 1 minute. Individual Crossing point (Cp) values were calculated using LC480 II software (Roche). Genomic DNA contamination was assessed by comparing expression values for splice-junction spanning and intergenic *TFCP2* assays. Additional data relating to MIQE criteria can be found in Additional file 1: Table S1.

### Statistical methods

All analysis was performed using R, version 2.15. Standard methods were used to estimate means, SD’s, and correlations. Individual gene expression values were normalized to HNRNPF and TFCP2; normalization within the GES occurs at the meta-gene level through the use of ratios and low variability genes such as HNRNPF and TFCP2. Details regarding the derivation and validation of the GES have previously been published [4,15]. Where appropriate, paired or unpaired t-tests were used to compare different processing conditions with a reference condition. Gene expression pre-processing and score calculations were performed as previously described [4].

## Results

### Initial performance assessment of RSM in small-animal studies

**Figure 1:**
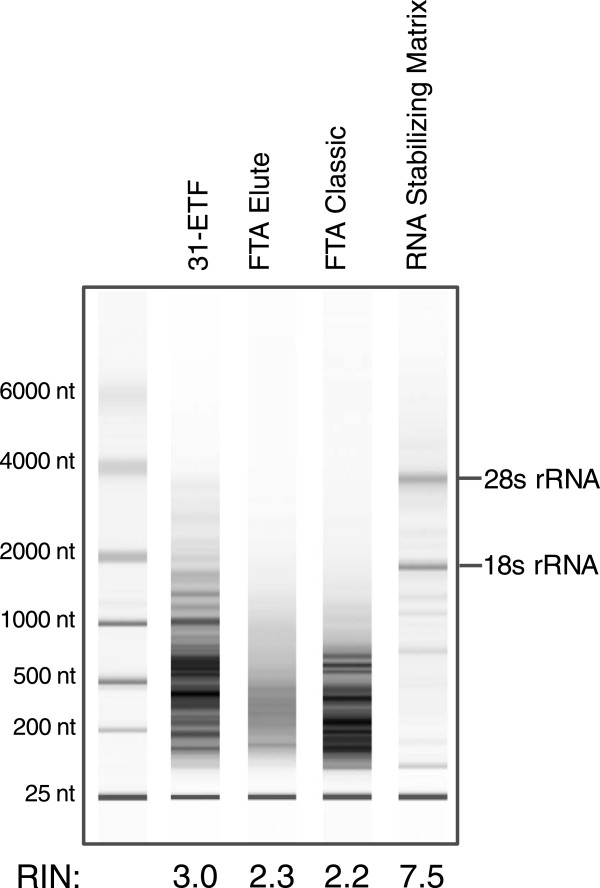
**Electropherogram of total rat RNA obtained from dried blood spots stored for 11 days at room temperature on various filter papers.** Pico Ladder standards are shown in the left lane. Agilent Bioanalyzer RNA Integrity Number (RIN) scores are shown at the bottom.

We compared the performance of conventional dried blood spot technologies [17] to RSM filter paper initially using a rat model. Fifty microliters of tail vein blood was spotted directly onto various filter paper matrices and stored for 11 days at room temperature. The integrity of total RNA extracted from RSM filter paper was compared to that from FTA paper, as well as untreated Whatman 31-ETF paper (Figure 1). RNA obtained from FTA and 31-ETF papers had RNA Integrity Number (RIN) scores of ≤ 3, with no visible 18S or 28S RNA bands present (Figure 1). In contrast, RNA from RSM paper had a RIN score of 7.5, with distinct 18S and 28S bands observed (Figure 1).

### Performance of RSM with human blood under different storage conditions

RNA integrity, yield and Real Time-qualitative PCR (RT-qPCR) performance were compared from human blood samples collected on RSM filter paper and PAXgene tubes subjected to 3 different storage conditions: a baseline arm (18 hours at RT), 18 hours at 37°C, and 6 days at RT (Additional file 2: Figure S1).

#### *-RNA integrity and yield*

**Figure 2:**
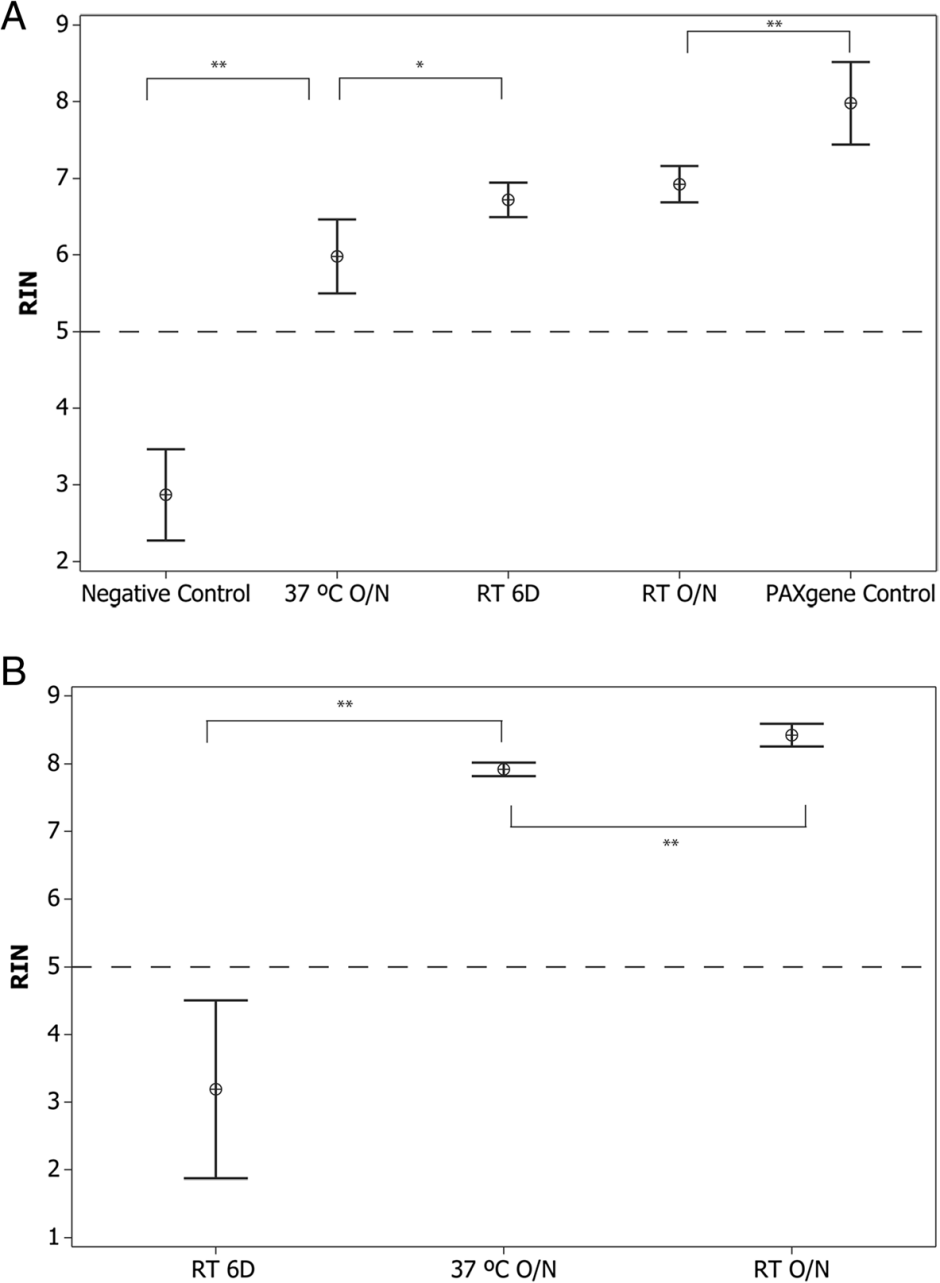
**RIN scores of RNA obtained from RSM or PAXgene samples stored under various conditions. (A)** RIN scores for RNA obtained from either 25 subjects (PAXgene Control, RT O/N) or a subset of 10 subjects (37°C O/N, RT 6D, Negative Control). Mean values ± 95% CI are shown. ** = p < 0.001, * = p 0.002 by paired t-test. **(B)** RIN scores (y axis) for RNA obtained from pooled PAXgene control samples subjected to various conditions. 10 replicates per condition were used. Mean values ± 95% CI are shown. ** = p < 0.001 by paired t-test. Dashed lines indicate minimum acceptable RIN for downstream gene expression analysis.

The baseline arm of this study utilized 25 healthy subjects and consisted of whole blood samples spotted on RSM and 31-ETF paper or collected in PAXgene tubes and stored for 18 hours at room temperature (baseline condition, RT; ~25°C). RIN scores for the RNA obtained from the PAXgene tubes were slightly higher than those obtained from RSM (mean RIN ±95% CI: 7.98 ± 0.54 vs. 6.92 ± 0.24 respectively, p < 0.001; Figure 2A, Additional file 3: Figure S2A, B); mean yields were higher from the PAXgene tubes (6.58 ng/μl vs. 4.79 ng/μl whole blood, respectively, p < 0.001) RNA isolated from untreated 31-ETF paper showed significant degradation (2.87 ± 0.11; Figures 1, 2A, Additional file 3: Figure S2C) and decreased mean yield versus baseline (2.34 ng/μl, p < 0.001).

To understand the impact of both time and temperature on RNA stored on RSM filter paper, RNA was extracted from dried blood spots stored for 18 hours at 37°C and 6 days at RT. A subset of 10 samples from the baseline arm was used for this 2-arm study (Additional file 2: Figure S1). RNA obtained from RSM stored for 18 hours at 37°C had slightly lower RIN scores compared to baseline (5.98 ± 0.48 vs. 6.92 ± 0.24 respectively, p < 0.001, Figure 2A, Additional file 3: Figure S2D) and lower mean yield compared to baseline (4.46 ng/μl, p > 0.05) whereas the RNA obtained from RSM stored for 6 days at RT had RIN scores comparable to baseline samples (6.72 ± 0.23, Figure 2A, Additional file 3: Figure S2E); with slightly higher mean yields versus baseline (5.4 ng/μl, p > 0.05) Paxgene RNA stored at 37°C versus room temperature for 18 hours also showed slightly lower RIN values (7.92 ± 0.1, 8.42 ± 0.17 respectively, p < 0.001; Figure 2B, Additional file 3: Figure S2F, G) and lower mean yield compared to baseline (4.86 ng/μl, p < 0.001); whereas PAXgene RNA stored for 6 days at RT displayed significant degradation (3.19 ± 1.32, p < 0.001; Figure 2B, Additional file 3: Figure S2H) and lower mean yield versus baseline (5.78 ng/μl, p < 0.05).

#### *-RT-qPCR*

**Figure 3:**
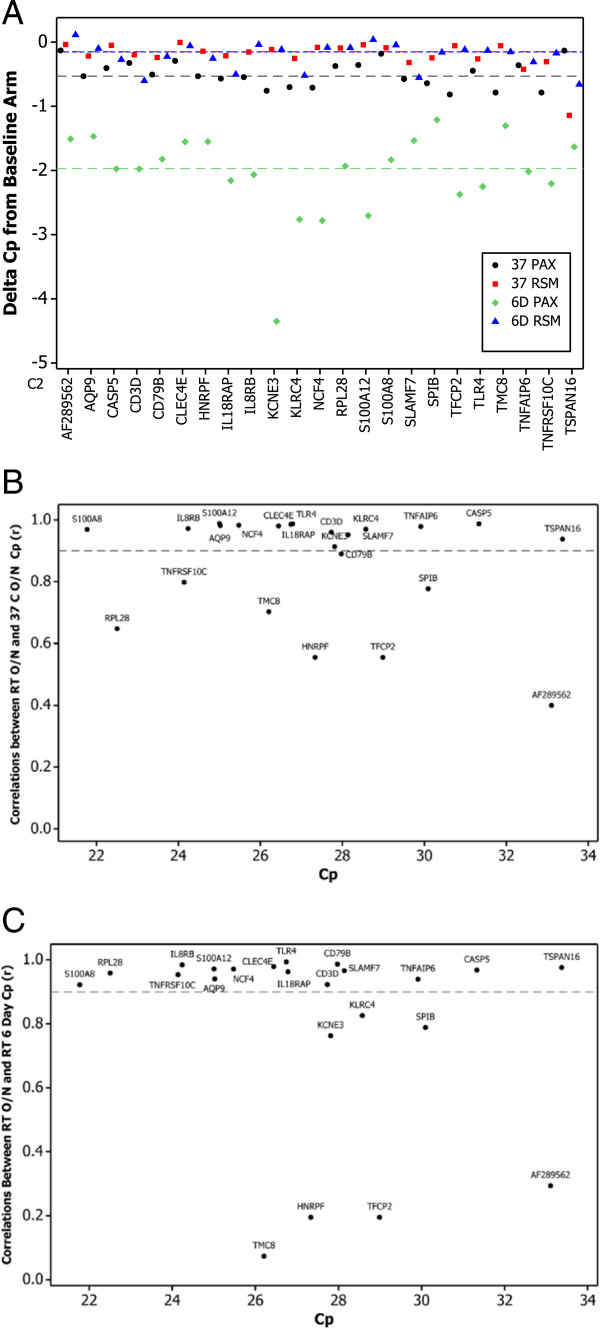
**Delta Cps and Pearson correlation values (r) obtained by comparing expression levels of 23 genes in the baseline arm (RT O/N) versus 37°C O/N and RT 6D arms. (A)** The delta Cp for each gene, by condition, is shown on the Y axis; genes are shown on the X axis. Black • = PAXgene, 37 C; Red ■ = RSM, 37 C; Green ♦ = PAXgene, 6 days; Blue ▲ = RSM, 6 day. Horizontal lines represent the median delta Cp of all genes for each condition; colors of lines are same as above. **(B)** Pearson correlation values (r) derived by comparing RT O/N to 37°C O/N (y axis) vs. Cp values (x axis) for RSM; dashed line denotes r value of 0.9 **(C)** Pearson correlation values (r) derived by comparing RT O/N to RT 6D (y axis) vs. Cp values (x axis) for RSM; dashed line denotes r value of 0.9.

To evaluate the RNA isolated under the various conditions with a functional test, 23 genes used in a clinically validated, gene expression-based diagnostic test for obstructive coronary artery disease [4] were assayed by RT-qPCR. Overall, only slight changes in gene expression levels were observed from RSM paper stored at 37°C or 6 days compared to the baseline arm, with a median delta Crossing point (Cp) of 0.15 observed for both comparisons (Figure 3A). Fifteen of the 23 genes tested showed no significant difference in expression levels (Cp values) between the 3 arms (Table 1). Two genes (CD3D, TLR4) showed a significant shift in Cp values in the 37°C arm whereas 7 genes (CD3D, IL18RAP, KLRC4, NCF4, RPL28, TNFAIP6, TNFRSF10C) showed significant shifts in the 6-day arm (Table 1), with only CD3D affected significantly in both arms. In comparison, the median delta Cp between the baseline arm and 37°C was slightly larger for PAXgene samples relative to RSM (0.53 vs. 0.15 Cp respectively; Figure 3A), and noticeably larger for PAXgene when compared to the 6-day arm (1.97 vs. 0.15 Cp; Figure 3A). Thus, for the 23 genes analyzed, mean gene expression from RSM paper was less sensitive to elevated temperature or prolonged storage compared to PAXgene-stabilized RNA.

**Table 1:** Time and temperature induced changes in the expression levels of 23 genes relative to the baseline condition

	**Baseline vs. 37°C**			**Baseline vs. 6 Day**		
**Gene**	**Estimate**	**95% CI**	P*	**Estimate**	**95% CI**	P*
AF289562	-0.371	0.739	0.285	-0.193	0.692	0.537
AQP9	0.044	0.065	0.159	-0.061	0.076	0.102
CASP5	-0.047	0.088	0.259	0.022	0.138	0.728
CD3D	-0.068	0.051	**0.015**	0.087	0.067	**0.017**
CD79B	-0.050	0.147	0.460	0.096	0.161	0.207
CLEC4E	0.017	0.054	0.499	0.029	0.049	0.205
HNRPF	-0.007	0.059	0.806	0.039	0.074	0.257
IL18RAP	0.006	0.065	0.843	0.082	0.064	**0.018**
IL8RB	0.017	0.055	0.495	-0.023	0.075	0.499
KCNE3	0.078	0.080	0.055	0.159	0.299	0.254
KLRC4	0.051	0.098	0.270	0.183	0.168	**0.036**
NCF4	0.022	0.038	0.227	0.060	0.030	**0.002**
RPL28	-0.040	0.087	0.326	-0.104	0.101	**0.044**
S100A12	-0.054	0.080	0.164	-0.051	0.074	0.154
S100A8	-0.021	0.061	0.462	-0.038	0.064	0.208
SLAMF7	0.001	0.082	0.981	0.070	0.092	0.117
SPIB	-0.023	0.213	0.812	-0.037	0.201	0.680
TFCP2	-0.011	0.101	0.809	-0.053	0.125	0.356
TLR4	0.072	0.044	**0.005**	-0.064	0.078	0.095
TMC8	-0.018	0.071	0.579	-0.025	0.132	0.671
TNFAIP6	0.031	0.110	0.540	-0.229	0.150	**0.008**
TNFRSF10C	-0.062	0.274	0.620	-0.285	0.284	**0.050**
TSPAN16	0.042	0.519	0.858	0.255	0.444	0.221

*p values < 0.05 are in bold.

Condition-induced shifts in gene expression levels may be uniform in magnitude and direction. To assess this, gene expression correlations (r) were calculated across both arms (Figure 3B, C). The majority of the genes showed strong correlations across the conditions evaluated. Five genes showed low correlations (r < 0.9) in both arms (SPIB, TMC8, AF289562, HNRPF, TFCP2; Figure 3B, C); 3 genes showed low correlations only in the 37°C arm (RPL28, CD79B, TNFRSF10C; Figure 3A) whereas 2 genes showed low correlation only in the 6-day arm (KLRC4, KCNE3; Figure 3B). The genes with lower correlations are known to have either lower expression levels that can lead to increased variability (e.g. AF289562) or, as previously reported, have lower biological variability in their expression levels, making them more sensitive to changes in conditions [16].

#### *-Gene Expression Score (GES)*

**Figure 4:**
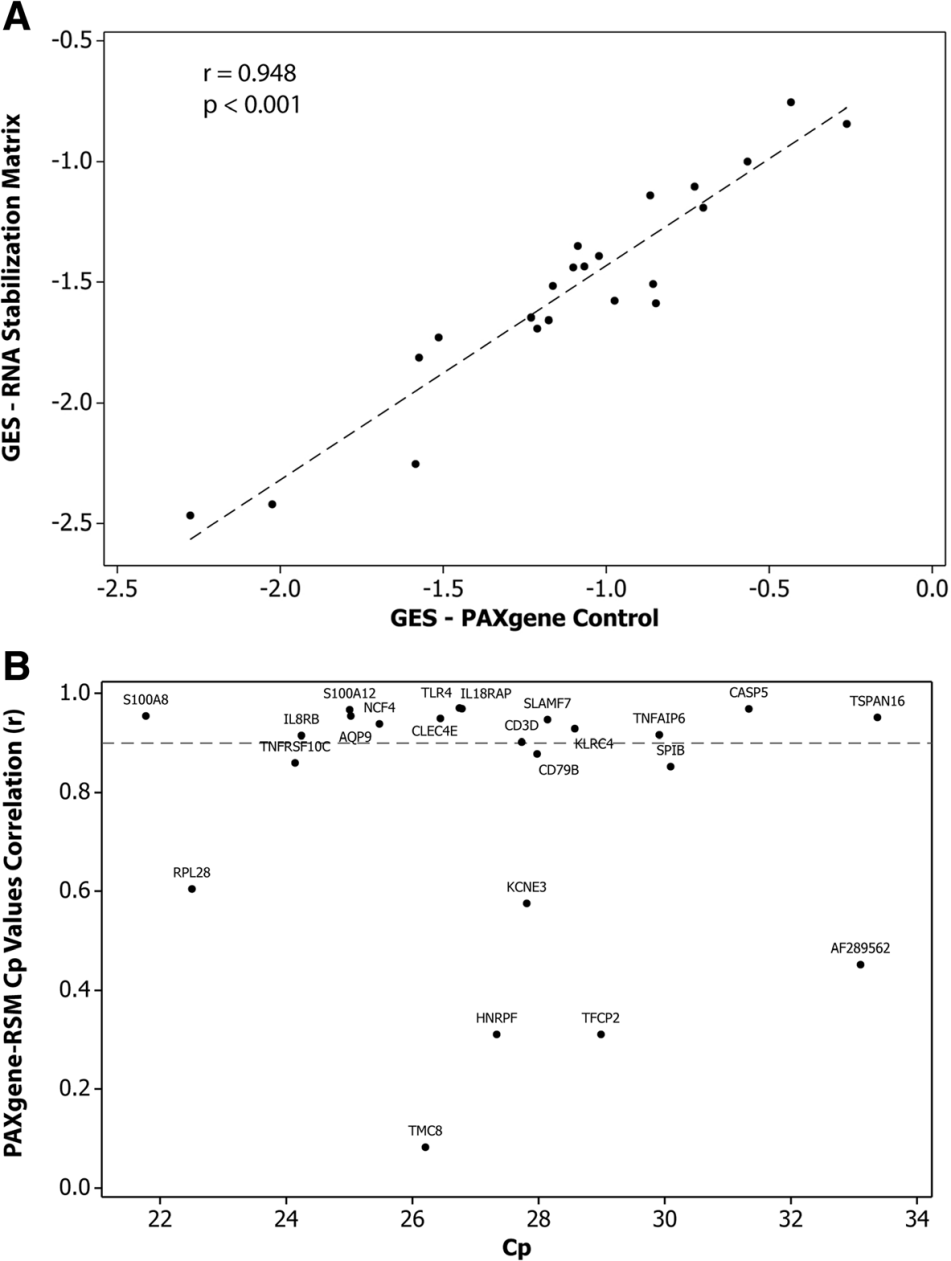
**Correlation of GES and expression levels of underlying 23 genes between RSM and PAXgene under baseline condition. (A)** GES for 25 subjects computed on RNA obtained from RSM (y axis) vs. PAXgene (x axis). **(B)** Pearson correlation values (r) derived by comparing RSM to PAXgene (y axis) vs. Cp values (x axis); dashed line denotes r value of 0.9.

To assess how RSM might perform in the context of a gene expression-based diagnostic test (Corus CAD), the GES was calculated from the samples obtained from different condition arms as well as from the PAXgene controls. The GES algorithm was developed and clinically validated using whole blood stabilized in PAXgene tubes [4,14]. Baseline arm samples collected via PAXgene tubes showed significant GES correlations with those collected in parallel onto RSM filter paper (r = 0.948, p < 0.001; Figure 4A). At the gene level, 15 genes showed strong correlations between the PAXgene and RSM methods, with 8 displaying weaker correlations (r < 0.9; CD79B, TNFRSF10C, SPIB, RPL28, KCNE3, AF289562, HNRNPF, TFCP2, TMC8; Figure 4B.)

Compared to the baseline arm, no significant change in GES were observed for RSM samples stored at 37°C nor in the samples stored for 6 days at RT (p > 0.05). Overall, the high correlation between RSM- and PAXgene-derived expression indicates that RSM could feasibly be used for such a diagnostic test with minimal adjustment to the predictive GES.

## Discussion

Solid, matrix-based based approaches have been successfully developed for the collection and storage of DNA, most notably for FTA cards. Use of FTA cards for the storage of RNA is most effective with cold temperature storage [17]. As such, we have developed and evaluated RSM, a matrix-based platform designed specifically for the collection and storage of RNA in whole blood at ambient temperature. Similar to previous findings with FTA cards, RNA is passively eluted from RSM by rehydration of the dried blood spot [17].

The baseline performance of RSM and subsequent effects of temperature and storage period were evaluated using a clinically validated and commercially available gene expression assay for the detection of obstructive coronary artery disease in non-diabetic patients (Corus CAD) [4]. The GES was derived using Ridge regression from 640 patients for whom real-time polymerase chain reaction gene expression data and angiographic data had been obtained [4]. The GES comprises expression values for 23 genes from peripheral blood cells in 6 terms as well as patient age and sex. Each term is composed of ratios of highly correlated genes representing a diverse set of inflammatory cell biology, including neutrophil apoptosis, neutrophil-to-lymphocyte ratio, and natural killer-cell activation.

The integrity of RNA obtained from RSM was relatively insensitive to elevated temperature or extended storage, showing a slight decrease in RIN values compared to the baseline arm. Compared to RNA obtained from the PAXgene control arm, RIN from RSM scores were significantly lower but well above the conventional cut-off (RIN >5) for most RNA-based applications [18,19]. In support of this finding, mean gene expression values and calculated GES derived from the expression levels of the genes were highly correlated between RSM and PAXgene tubes in the baseline arm. PAXgene samples stored at elevated temperature or for a prolonged period showed less consistent results, similar to other published reports [20]. RNA obtained from tubes stored for 6 days at room temperature showed significantly lower RIN scores compared to RSM, while PAXgene samples stored at 37°C yielded higher RIN values but RNA yield from these tubes was much lower than baseline (data not shown). At the single-gene level, the expression of a relatively small number of genes was significantly affected by either temperature or storage time on RSM (Table 1; Figure 3A). However, the global shift in gene expression was more pronounced in RNA obtained from PAXgene samples at 37°C or 6-day storage, indicating an increased sensitivity to temperature and time compared to RSM.

Relative to the baseline study, 18 genes showed high correlation in both elevated time and temperature arms, with five genes demonstrating lower correlations (r < 0.9, Figure 3B, C). These lower correlations may be driven in part by lower overall biological variability in the expression levels of these genes [16]. Notably, these specific genes also showed lower correlation with PAXgene-derived expression levels (Figure 4B). However, from a global perspective, the calculated GES displayed good correlation between RSM and PAXgene derived RNA (r = 0.948, p < 0.001; Figure 4A), demonstrating that RSM could feasibly be used for such a diagnostic test with minimal adjustment to the GES.

An increasing number of blood based gene-expression signatures for the detection of disease have recently been developed [4,5,21]. An integral part in the development and clinical use of such tests is the ability to reproducibly obtain high quality RNA. To this end, a number of tube-based methods containing RNA-preservative solutions have been successfully employed [8,9]. Tube-based collection methods do however pose a number of limitations. Clinical sites may not have access to a phlebotomist, making sample collection a challenge. In addition, transport of blood in tubes may require temperature-controlled environments, which can add substantial costs. Finally, sample volumes are typically in the milliliter range, an amount that may be impractical to obtain in certain clinical scenarios (e.g. work with neonates or small animals) [11,12]. Some efforts have been made to miniaturize collection volumes using PAXgene preservative, however it is unclear how easily these methods would translate to wider scale use [11,12,22]. In these regards, dried blood spot technology such as RSM offers significant advantages.

This study is not without certain limitations. For simplicity, whole blood was collected in EDTA tubes prior to manual application onto the RSM filter paper; an automated methodology for directly applying blood onto this matrix needs to be evaluated and developed. Although RNA derived from RSM was of relatively high integrity, the performance of such RNA was only evaluated by RT-qPCR, a modality which can tolerate significant amounts of RNA degradation especially if small amplicons are used [23]. Other modalities measuring gene expression levels such as microarrays or RNAseq could be more sensitive to RNA integrity but this remains to be determined. Additionally, a limited number of potential transport and storage conditions were evaluated; further studies will need to be performed if this media is to be considered as a long-term storage option. As the bead-based method utilized to purify RNA was optimized for larger transcripts, we were unable to assess the ability of RSM to stabilize smaller RNA species such as miRNA; we hope to address this limitation in future studies. Finally, RSM was produced in a research environment. Lot-to-lot reagent variability has been shown to contribute to variability in gene expression-based tests; further work needs to be performed on commercial-grade RSM to evaluate lot-to-lot differences [16].

## Conclusions

We have evaluated a dried blood spot matrix optimized for collecting and preserving high-quality RNA, comparing RNA integrity and gene expression levels relative to PAXgene blood collection tubes drawn in parallel. We have demonstrated that comparable results are obtained between RSM filter paper and PAXgene blood collection tubes under overnight sample storage conditions, and RSM performance exceeds PAXgene results with longer-term room temperature storage. Although the current study was performed on a relatively small set of healthy subjects, the results indicate that with further development an RSM-like approach may provide an attractive, dry-state method for obtaining and storing RNA from whole blood samples. Studies evaluating the performance of RSM in a larger clinical setting using a refined collection methodology are warranted, and may provide additional evidence supporting the use of RSM in real-world clinical settings.

## Competing interests

HT, PB, SR and JW are employees of CardioDx, Inc. and have equity interests and/or stock options in CardioDx. BL, WA and EK are employees of GE Healthcare and have equity interests and/or stock options in GE; BL and EK have filed patent applications on behalf of GE (US20130289265, US20130289257, and US20130338351). Corus CAD and FTA are registered trademarks of CardioDx and GE Healthcare, respectively. This study was jointly funded by CardioDx, Inc. and GE Healthcare.

## Authors’ contributions

HT performed all of the experiments evaluating the performance of RSM and PAXgene tubes using human blood in addition to providing data analysis. PB contributed to the experimental design and provided statistical analysis. EK performed the initial analysis using rat blood. BL and WA prepared the RSM and participated in QC analysis and performance testing of RSM. EK and JW conceived, designed and coordinated the study, and in addition co-wrote the manuscript. SR helped critically revise the manuscript. All authors read and approved the final manuscript.

## Pre-publication history

The pre-publication history for this paper can be accessed here:

http://www.biomedcentral.com/1472-6890/14/22/prepub

## Supplementary Material

Additional file 1: Table S1 MIQE data for the 23 genes used to generate the GES. Click here for file

Additional file 2: Figure S1 Experimental flow for 25 subjects used in the PAXgene vs. RSM comparison (A) and for the 10 PAXgene pool control samples (B). Click here for file

Additional file 3: Figure S2 Agilent Bioanalyzer traces of the RNA isolated from the 25 subjects collected in PAXgene tubes and stored overnight at RT (A); 25 subjects collected on RSM paper and stored overnight at RT (B); 10 subjects collected on 31-ETF paper and stored overnight at RT (C); 10 subjects collected on RSM and stored overnight at 37°C (D); 10 subjects collected on RSM and stored for 6 days at RT (E); 10 PAXgene pool controls stored overnight at RT (F); 10 PAXgene pool controls stored overnight at 37°C (G); 10 PAXgene pool controls stored for 6 days at RT. Click here for file

## Requests for material

Requests by researchers for RSM by should be made to EK.
