# Supplementary figures and images for: Evaluation of a solid matrix for collection and ambient storage of RNA from whole blood
Source: BMC Clin Pathol. 2014 May 13;14:22. doi: 10.1186/1472-6890-14-22 (PMC4030268; doi:10.1186/1472-6890-14-22)

Supplementary Figure 1

(A)

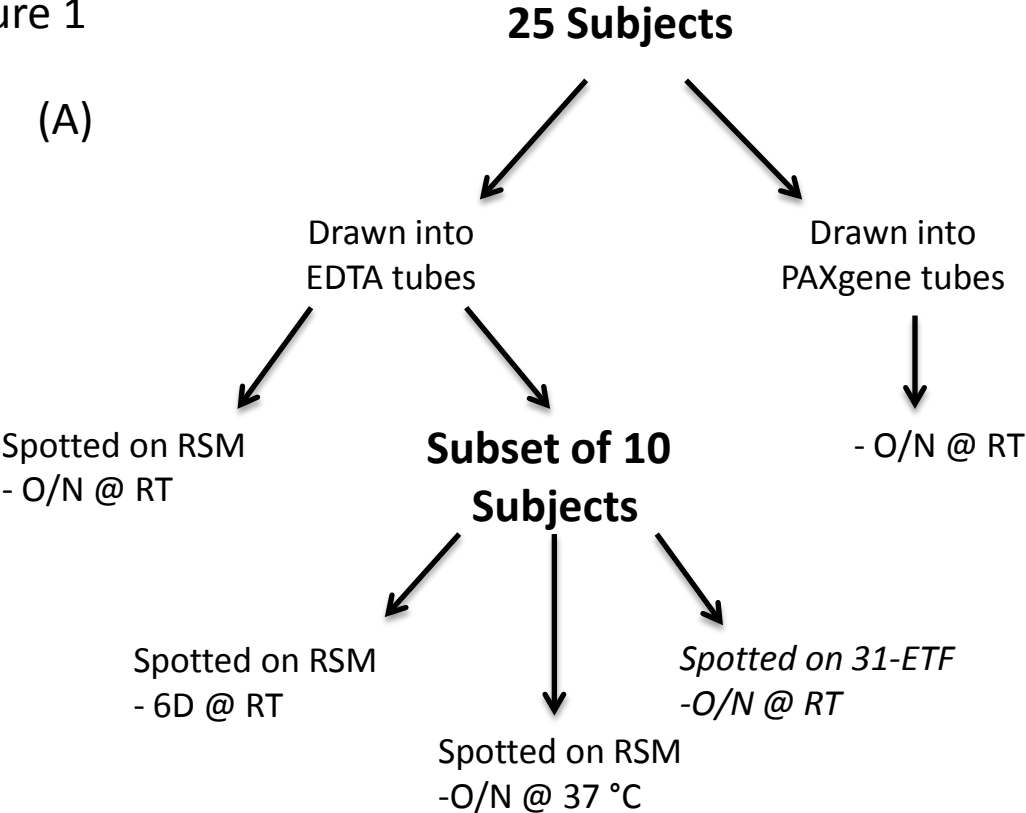

(B)

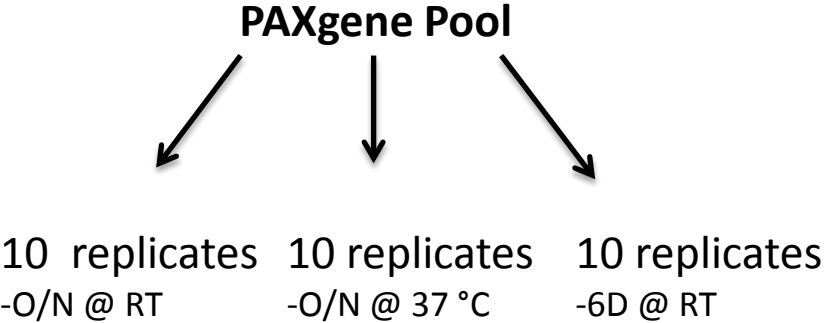

Supplement: Additional file 2: Figure S1 — Experimental flow for 25 subjects used in the PAXgene vs. RSM comparison (A) and for the 10 PAXgene pool control samples (B). [file 1472-6890-14-22-S2.pdf]
