# Supplementary material for: Evaluation of a solid matrix for collection and ambient storage of RNA from whole blood
Source: BMC Clin Pathol. 2014 May 13;14:22. doi: 10.1186/1472-6890-14-22 (PMC4030268; doi:10.1186/1472-6890-14-22)

(A) PAXgene Baseline

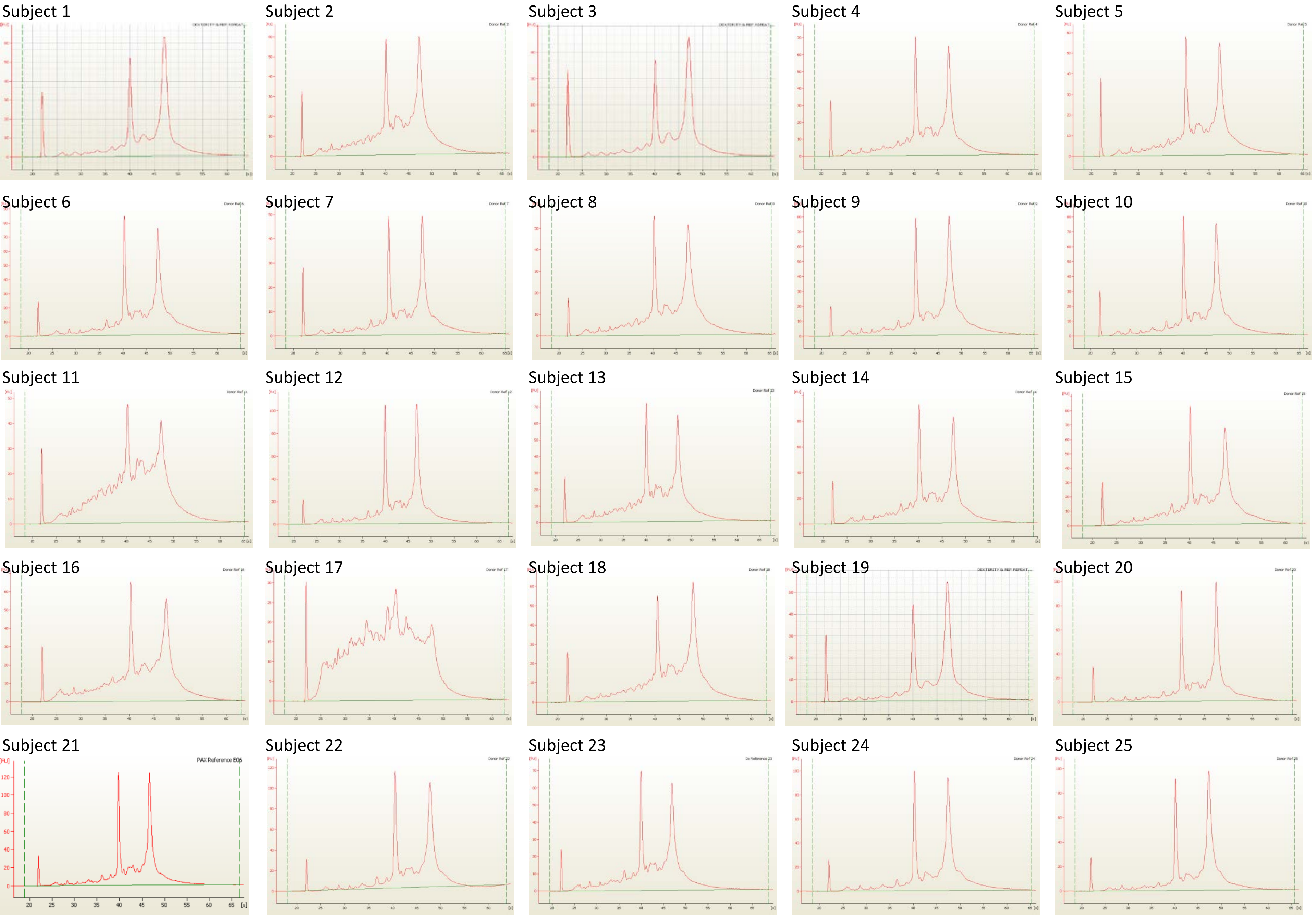

(B) RSM Baseline

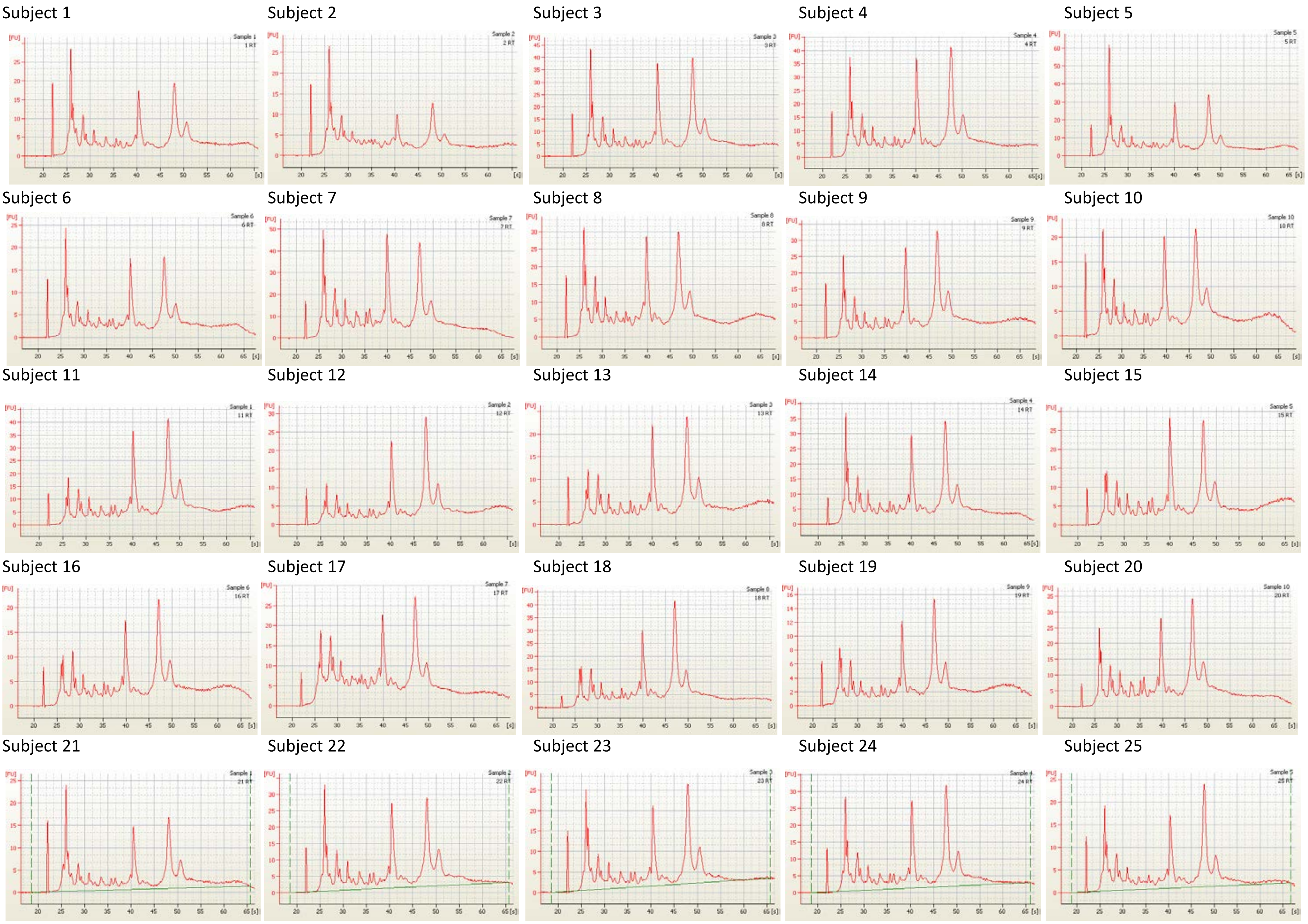

(C) 31-ETF Baseline

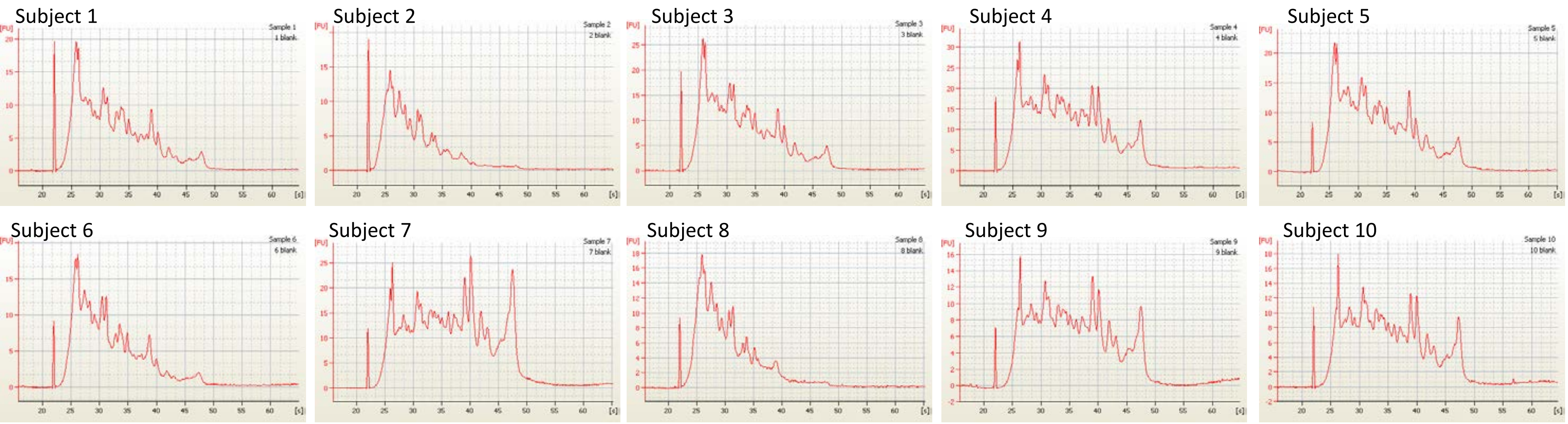

(D) RSM 37 °C

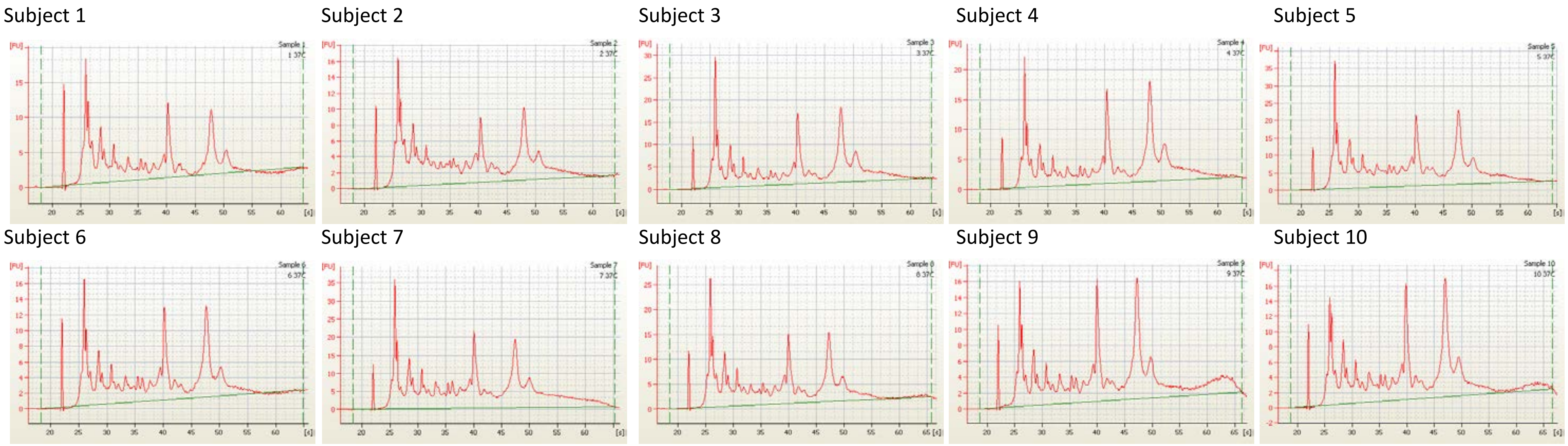

(E) RSM 6 Days

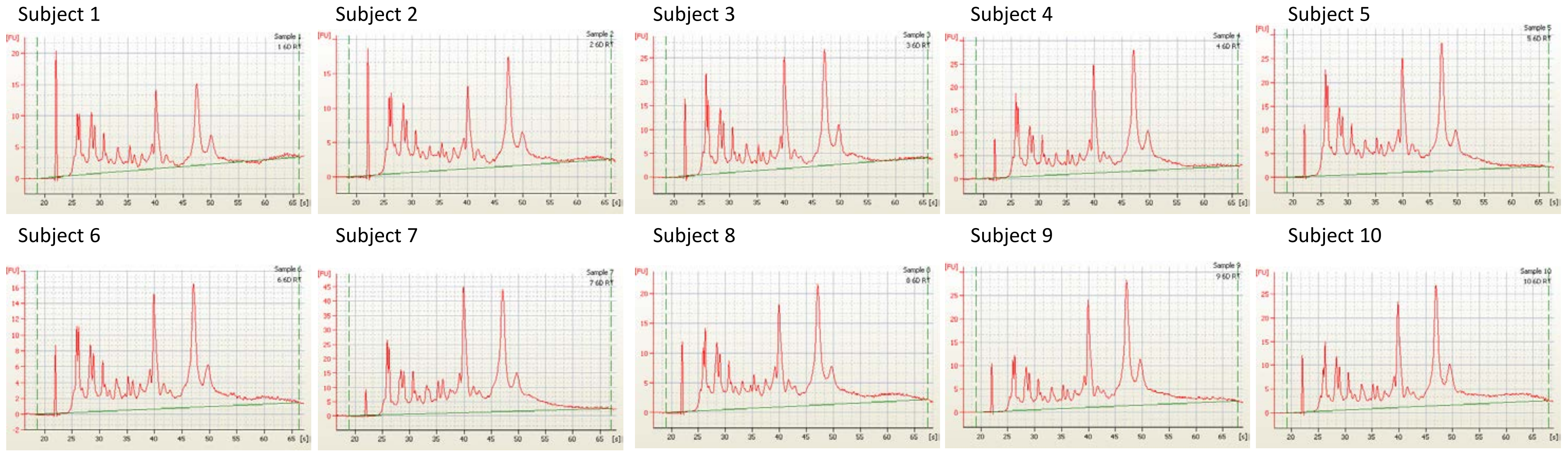

(F) PAXgene Controls Baseline

Replicate 1

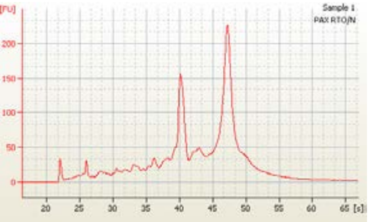

Replicate 2

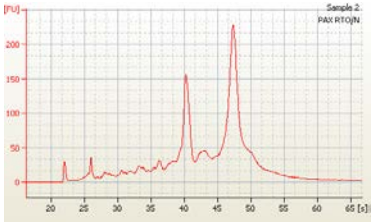

Replicate 3

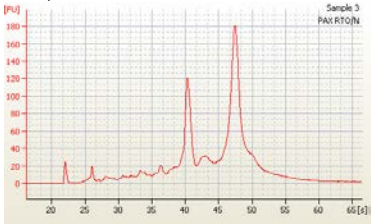

Replicate 4

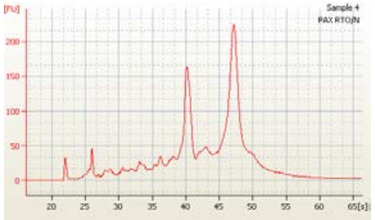

Replicate 5

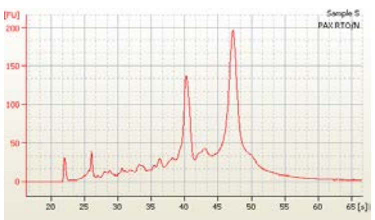

Replicate 6

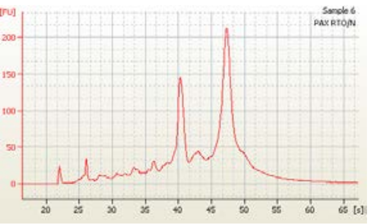

Replicate 7

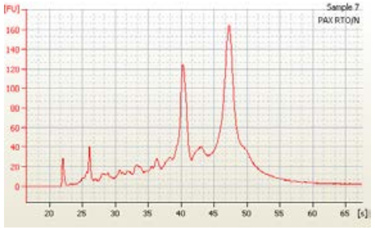

Replicate 8

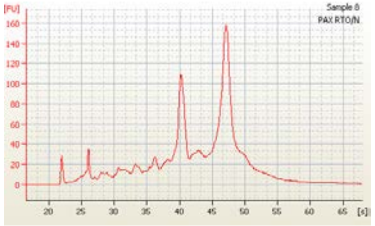

Replicate 9

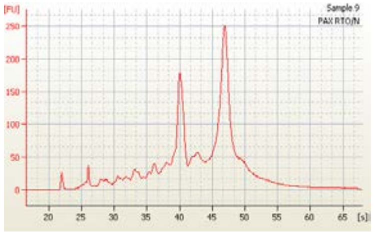

Replicate 10

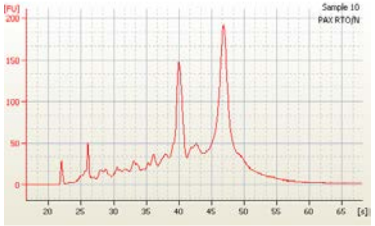

(G) PAXgene Controls 37 °C

Replicate 1

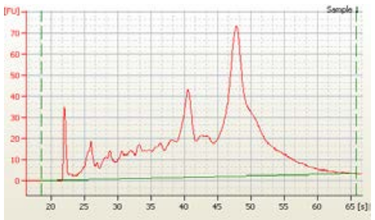

Replicate 2

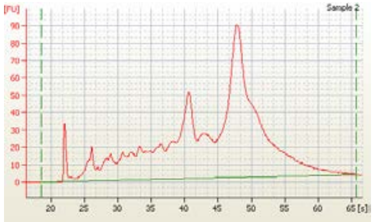

Replicate 3

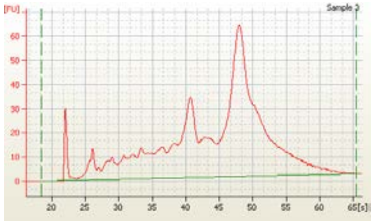

Replicate 4

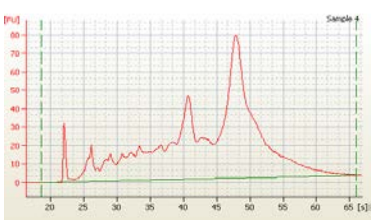

Replicate 5

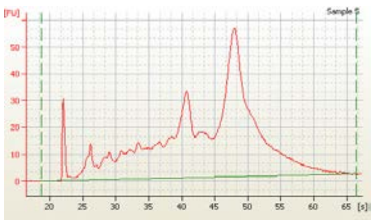

Replicate 6

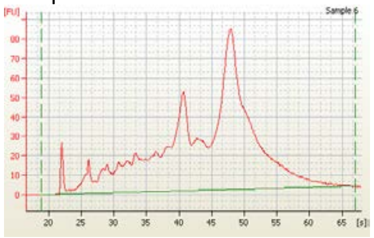

Replicate 7

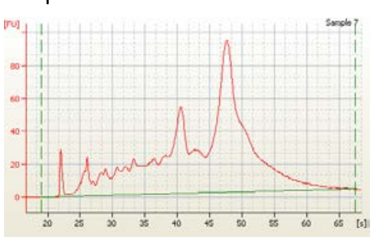

Replicate 8

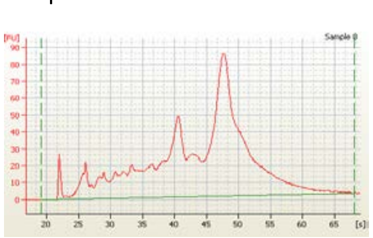

Replicate 9

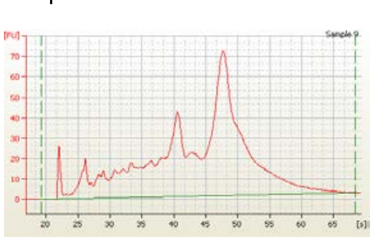

Replicate 10

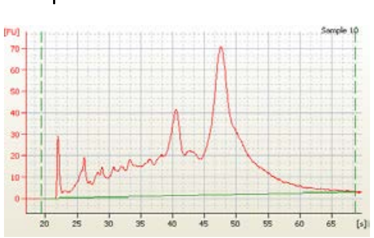

(H) PAXgene Controls 6 Days

Replicate 1

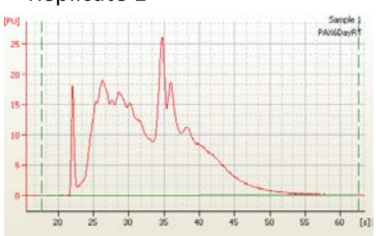

Replicate 2

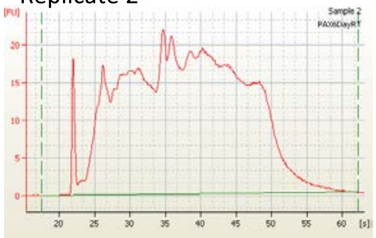

Replicate 3

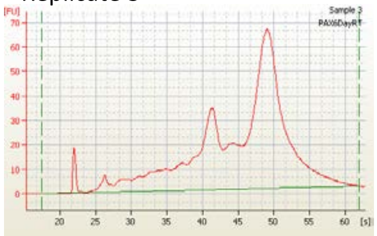

Replicate 4

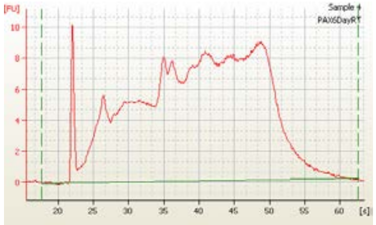

Replicate 5

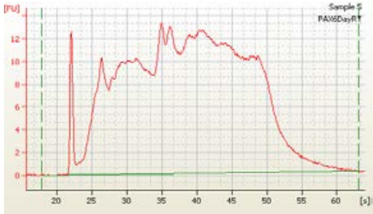

Replicate 6

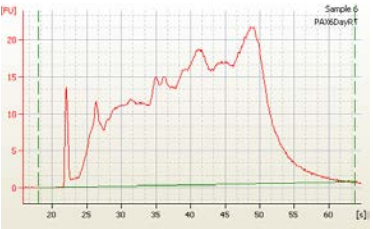

Replicate 7

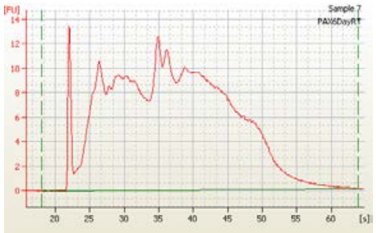

Replicate 8

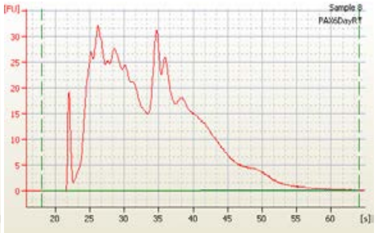

Replicate 9

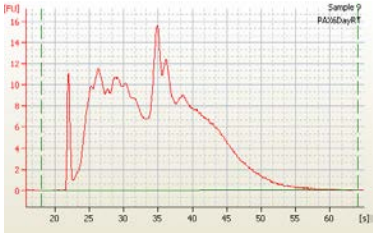

Replicate 10

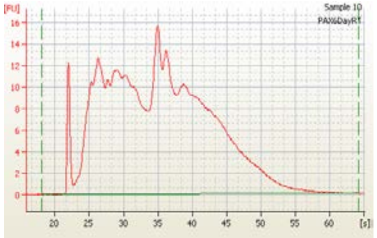

Supplement: Additional file 3: Figure S2 — Agilent Bioanalyzer traces of the RNA isolated from the 25 subjects collected in PAXgene tubes and stored overnight at RT (A); 25 subjects collected on RSM paper and stored overnight at RT (B); 10 subjects collected on 31-ETF paper and stored overnight at RT (C); 10 subjects collected on RSM and stored overnight at 37°C (D); 10 subjects collected on RSM and stored for 6 days at RT (E); 10 PAXgene pool controls stored overnight at RT (F); 10 PAXgene pool controls stored overnight at 37°C (G); 10 PAXgene pool controls stored for 6 days at RT. [file 1472-6890-14-22-S3.pdf]
